# The Multifunctional SMC5/6 Complex in Genome Stability, Antiviral Restriction, and Human Disease

**DOI:** 10.3390/cimb48090880

**Published:** 2026-08-30

**Authors:** Yaqing Zhang, Weijie Lai, Jiale Song, Chubai Qiu, Ru Yan, Qi Zhao, You Yu

**Affiliations:** 1 Department of Cardiology, the Second Affiliated Hospital, Zhejiang University-University of Edinburgh Institute, Zhejiang University School of Medicine, Zhejiang University, Hangzhou 310058, China; yaqing.24@intl.zju.edu.cn (Y.Z.); weijie.23@intl.zju.edu.cn (W.L.); jiale.22@intl.zju.edu.cn (J.S.); chubai.22@intl.zju.edu.cn (C.Q.); ru.22@intl.zju.edu.cn (R.Y.); qi1.24@intl.zju.edu.cn (Q.Z.); 2 Edinburgh Medical School, College of Medicine and Veterinary Medicine, The University of Edinburgh, Edinburgh EH8 9YL, UK; 3 ZJE Cross Lung Research Alliance, International Joint Center for Biomedical Sciences, Zhejiang University, Haining 314400, China

**Keywords:** SMC5/6 complex, genome stability, DNA damage repair, replication stress, antiviral restriction, viral evasion, developmental disorders, cancer

## Abstract

The Structural Maintenance of Chromosomes 5/6 (SMC5/6) complex is a key regulator of genome stability that participates in the recognition, stabilization, and processing of DNA intermediates generated during DNA replication and homologous recombination. Recent advances in structural and biochemical studies have revealed the architecture of the SMC5/6 complex and elucidated the molecular mechanisms by which ATPase activity, DNA-binding modules, and SUMO-and ubiquitin-mediated regulatory pathways cooperate to maintain genome integrity. Extensive evidence has further demonstrated that SMC5/6 functions as a broad-spectrum antiviral restriction factor by recognizing and silencing episomal viral genomes, whereas diverse viruses have evolved strategies to evade or counteract its activity. In addition, dysfunction of the SMC5/6 complex has been associated with cancer, developmental disorders, and genome instability syndromes. This review summarizes recent advances in the structure, molecular mechanisms, antiviral functions, and disease relevance of the SMC5/6 complex, providing a basis for a deeper understanding of its diverse biological functions and potential applications.

## 1. Introduction

Genome stability is essential for the faithful transmission of genetic information, normal cell proliferation, and the maintenance of organismal homeostasis in eukaryotes. Genomic instability, the accumulation of DNA damage, and chromosomal abnormalities are frequently associated with developmental defects, tumorigenesis, immune dysregulation, and other pathological conditions [1]. The Structural Maintenance of Chromosomes (SMC) protein family comprises a group of evolutionarily conserved proteins that play central roles in chromosome organization and genome maintenance [2]. Considerable advances have been achieved in elucidating the structural organization and biological functions of the cohesin and condensin complexes. Cohesin plays essential roles in sister chromatid cohesion, higher-order chromatin organization, and transcriptional regulation, whereas condensin is required for chromosome condensation and the maintenance of chromosome architecture during mitosis [3]. The eukaryotic SMC5/6 complex is composed of core structural subunits (SMC5 and SMC6) and non-SMC auxiliary subunits. These auxiliary subunits include Nse1 (NSMCE1), Nse2/Mms21 (NSMCE2), Nse3 (NSMCE3), Nse4 (NSMCE4A), as well as the Nse5/6 regulatory subcomplex. In humans, this subcomplex has expanded into two paralogous modules: SIMC1 and SLF1 (both orthologs of yeast Nse5), each associating with the Nse6 ortholog SLF2. In comparison, the molecular mechanisms and biological functions of the eukaryotic SMC5/6 complex remain less well characterized. Nevertheless, increasing evidence suggests that SMC5/6 participates in a broader range of cellular processes than previously appreciated, highlighting its multifaceted roles in maintaining genome stability and cellular homeostasis.

Studies to date have demonstrated that, in addition to its established roles in DNA replication, DNA repair, and chromosome maintenance, the SMC5/6 complex functions as a host restriction factor that recognizes and silences episomal viral genomes, thereby contributing to antiviral defense [4]. Dysregulation of the complex has also been linked to a variety of human diseases, including cancer, developmental disorders, and genome instability syndromes [5,6]. Given the substantial advances in this field over recent years, this review summarizes current knowledge of the structural organization, molecular regulatory mechanisms, antiviral activities and disease associations of the SMC5/6 complex, with the aim of advancing our understanding of its multifaceted biological functions and informing future mechanistic studies and potential applications.

## 2. Structural Organization of the SMC5/6 Complex and Its Comparison with Other SMC Family Members

**Figure 1:**
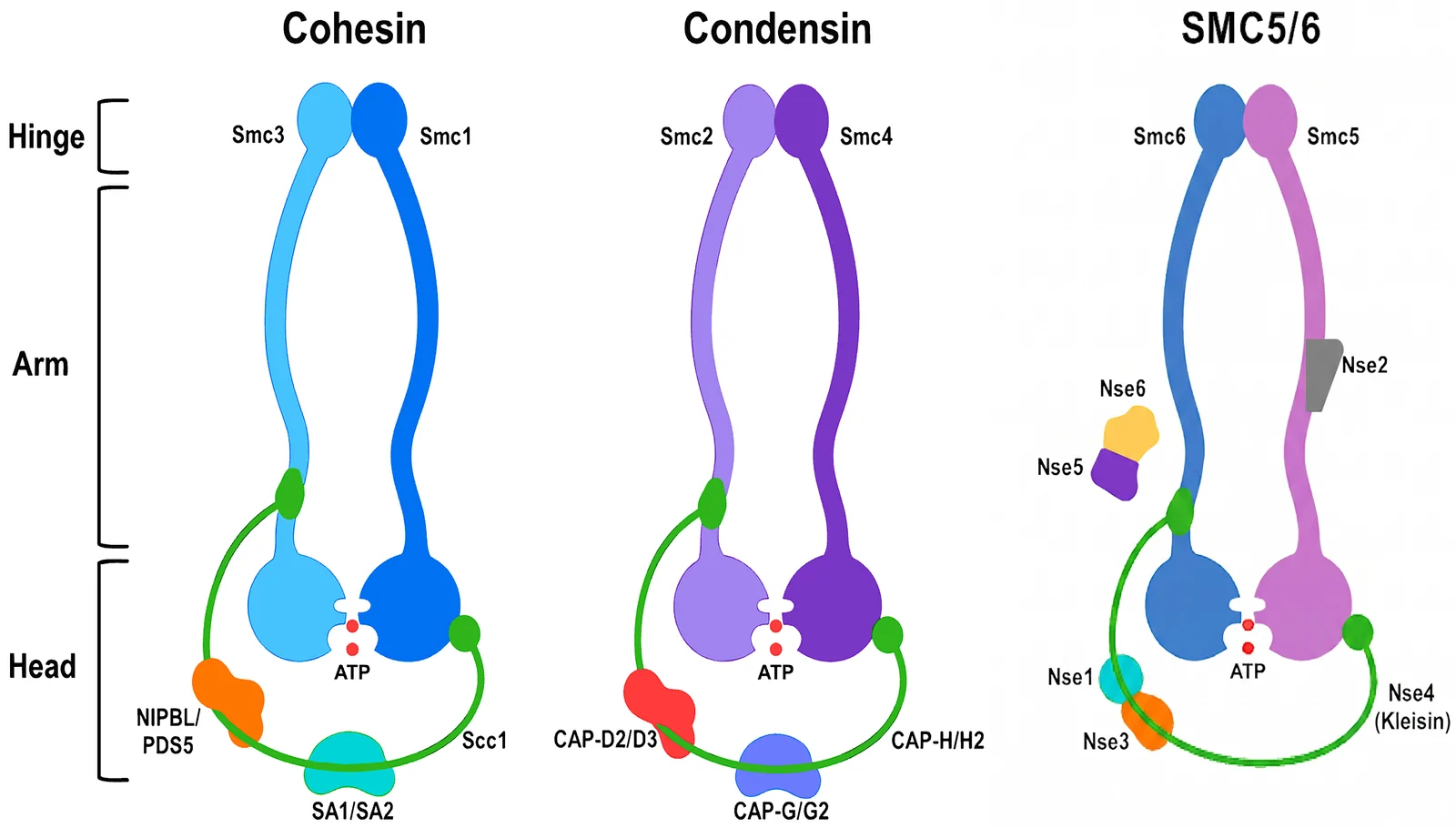
Comparison of the structural organization of Cohesin, Condensin, and SMC5/6 complexes. The conserved head–arm–hinge architecture of three eukaryotic Structural Maintenance of Chromosomes (SMC) complexes is shown: Cohesin, Condensin, and SMC5/6. Each complex contains two SMC ATPase subunits that dimerize through their hinge domains and extend into long coiled-coil arms connected to ATP-binding head domains. Cohesin consists of Smc1–Smc3, the kleisin subunit Scc1/Rad21, and associated regulatory subunits including SA1/SA2, NIPBL, and PDS5. Condensin consists of Smc2–Smc4, the kleisin subunits CAP-H or CAP-H2, and HEAT-repeat subunits CAP-D2/CAP-D3 and CAP-G/CAP-G2. In contrast, SMC5/6 consists of Smc5–Smc6 and multiple non-SMC elements, including the kleisin Nse4, the KITE subunits Nse1–Nse3, the SUMO E3 ligase Nse2, and the regulatory Nse5–Nse6 subcomplex. The figure was created using Microsoft PowerPoint and Canva. ATP—adenosine triphosphate; CAP—chromosome-associated protein; HEAT—Huntingtin, elongation factor 3, protein phosphatase 2A, and TOR1; KITE—kleisin-interacting tandem winged-helix element; NIPBL—Nipped-B-like protein; Nse—non-SMC element; PDS5—precocious dissociation of sisters protein 5; Rad21—DNA repair protein Rad21; SA1/SA2—stromal antigen 1/2; Scc1—sister chromatid cohesion protein 1; SUMO—small ubiquitin-like modifier.

The Structural Maintenance of Chromosomes 5/6 (SMC5/6) complex is a conserved yet functionally specialized member of the eukaryotic SMC family [7,8]. Although SMC5/6 is evolutionarily conserved across eukaryotes, many foundational mechanistic insights were initially obtained from yeast models, whereas recent structural and cellular studies have further defined conserved and human-specific features in mammalian systems. Like Cohesin and Condensin, it is built on the canonical SMC architecture—ATPase “heads”, long antiparallel coiled-coils, and a hinge that dimerizes the SMC subunits into a V-shaped scaffold—where ATP binding and hydrolysis at the head domains drive conserved conformational transitions transmitted through the coiled-coil arms (Figure 1) [7,9,10]. However, SMC5/6 deploys this conserved chassis for local, context-dependent genome maintenance rather than large-scale chromosome organization, a specialization that is largely encoded by how its non-SMC element (NSE) subunits rewire DNA engagement and ATPase control [8,11,12].

Across eukaryotic systems, including budding yeast and fission yeast, Smc5 and Smc6 form a stable heterodimer via their hinge domains, while the head–neck regions serve as the principal assembly and DNA engagement hub [10,13]. Recent cryo-EM studies of budding yeast SMC5/6 complexes indicate that the Smc5–Smc6 arms can adopt juxtaposed, rod-like arrangements and switch to ATP-engaged states in which head engagement is achieved through ATP “sandwiching”, providing a structural framework for regulated DNA confinement [9,10,14]. Critically, unlike a bare SMC dimer, SMC5/6 is organized into a kleisin-centered head module in which the kleisin Nse4 bridges the Smc5 and Smc6 head-proximal regions and recruits the KITE partners Nse1-Nse3, a class of kleisin-interacting tandem winged-helix elements, as demonstrated initially in both budding yeast and fission yeast and further supported by structural studies of mammalian complexes (Figure 1) [10,13,15]. This Nse1–Nse3–Nse4 assembly contributes a major DNA-interacting interface and, in DNA-bound states, can form a clamp-like configuration around duplex DNA at the head region, consistent with a mode of DNA capture optimized for transient, structure-rich intermediates that arise during replication stress and recombination [11,14]. Two additional NSE modules further distinguish SMC5/6 from its SMC relatives. First, across eukaryotes, SMC5/6 uniquely embeds a SUMO E3 ligase, Nse2/Mms21, as a constitutive subunit positioned along the Smc5 coiled-coil arm. This architecture places post-translational signaling capacity within the mechanical core of the complex and provides a direct route by which DNA engagement and conformational state can tune SUMOylation outputs on chromatin [8,16]. Second, studies in budding yeast demonstrated that the Nse5–Nse6 subcomplex acts as a regulatory adaptor that contacts the head/neck region and gates productive ATPase engagement. Recent structural analyses of budding yeast further support a conserved DNA-dependent regulatory mechanism—an inhibited state in which Nse5/6 restrains head engagement and ATP hydrolysis, with suitable DNA substrates relieving this inhibition, thereby coupling ATPase productivity to appropriate DNA structures rather than enabling constitutive, chromosome-wide activity [9,17]. Together, these features support a concise mechanistic logic for SMC5/6 action: selective recognition of stress-associated DNA topologies, gated activation of ATPase-driven DNA confinement, and localized signaling via intrinsic enzymatic modules [11,12,16].

This wiring contrasts with the organizational logic of Cohesin and Condensin (Figure 1). Although all three complexes share the same head–hinge–arm scaffold, Cohesin and Condensin primarily support global chromosome architecture through more persistent DNA engagement, topological or pseudo-topological DNA capture, and large-scale loop organization, facilitated by dedicated loading and regulatory factors [2,7,18]. In Cohesin, the Smc1–Smc3 ring is closed by the kleisin Scc1/Rad21 and stably associated with the SA/STAG subunit, a HAWK-family HEAT-repeat protein. This architecture is further controlled by additional kleisin-associated HAWK proteins, including the Scc2/Scc4 loader complex, represented by NIPBL–MAU2 in metazoans, and PDS5-containing regulatory assemblies, which together regulate Cohesin loading, residence time, release, and loop-extruding activity [7,15]. Condensin follows a related HAWK-based organizational principle but uses distinct kleisin and HEAT-repeat subunits: Condensin I contains Smc2–Smc4, the kleisin CAP-H, and the HEAT-repeat subunits CAP-D2 and CAP-G, whereas Condensin II contains Smc2–Smc4, the kleisin CAP-H2, and the corresponding HEAT-repeat subunits CAP-D3 and CAP-G2 (Figure 1) [15]. These HAWK-associated architectures enable Cohesin and Condensin to operate as chromosome-scale organizers, supporting sister chromatid cohesion, chromatin domain formation, and mitotic chromosome compaction [2,7].

SMC5/6, in contrast, uses the conserved SMC scaffold in a more damage- and replication-stress-responsive manner [8,12]. Rather than relying on loading systems that support persistent chromosome-wide organization, SMC5/6 couples its activity to local chromatin environments shaped by DNA damage, replication stress, and recombination-associated DNA structures [8,11]. This stress-responsive deployment reflects its distinct architectural features, including KITE- rather than HAWK-associated kleisin organization, intrinsic SUMO ligase embedding, and Nse5/6-mediated ATPase gating [9,15,16]. Together, these differences reshape a conserved SMC chassis into a genome maintenance machine optimized for transient DNA intermediates more than for broad chromosome architectural organization [11,12].

## 3. Cellular and Molecular Mechanisms of the SMC5/6 Complex in Genome Stability

### 3.1. Structure-Coupled Post-Translational Signaling by the SMC5/6 Complex

**Figure 2:**
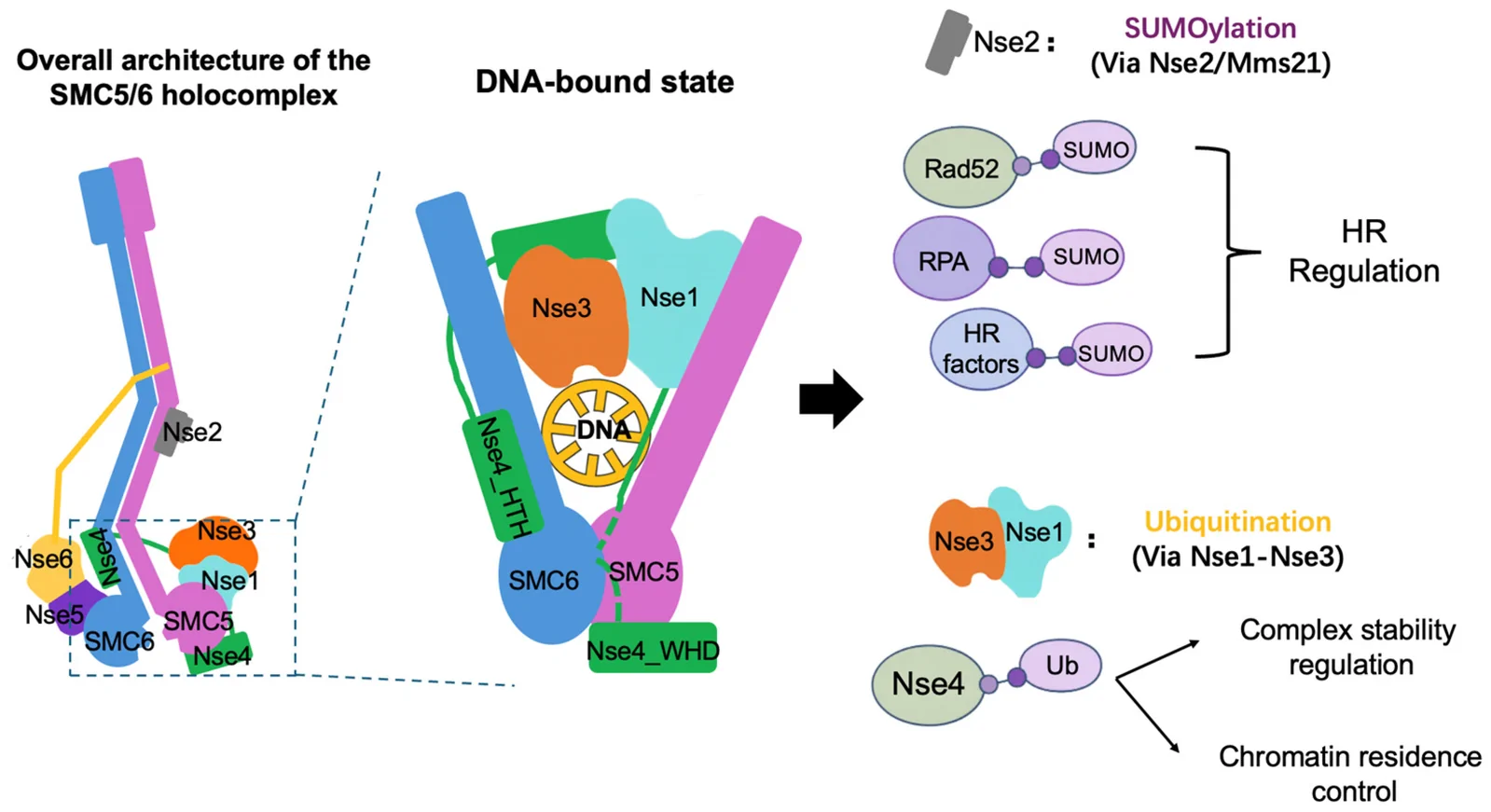
Structural organization of the SMC5/6 holocomplex and its post-translational regulatory functions. The SMC5/6 holocomplex consists of the core Smc5–Smc6 ATPase motor and multiple non-SMC elements (Nse1–Nse6), which collectively mediate DNA binding, structural maintenance, and genome stability control. Upon DNA engagement, SMC5/6 adopts an active DNA-bound conformation that coordinates replication stress responses and DNA damage repair. The SUMO E3 ligase subunit Nse2/Mms21 catalyzes SUMOylation of key homologous recombination (HR)-associated proteins, including Rad52, RPA, and additional HR factors, thereby fine-tuning HR progression, suppressing excessive recombination, and promoting controlled DNA repair outcomes. In parallel, the Nse1–Nse3 subcomplex mediates ubiquitination of SMC5/6-associated components, including Nse4, to regulate complex stability and chromatin residence dynamics at sites of genome stress. The figure was created using Microsoft PowerPoint and Canva. HR–homologous recombination; Mms21–methyl methanesulfonate sensitivity protein 21; Nse–non-SMC element; RPA–replication protein A; SMC–Structural Maintenance of Chromosomes; SUMO–small ubiquitin-like modifier; Ub–ubiquitin.

Structural and biochemical studies on yeast-derived and reconstituted recombinant SMC5/6 complexes have demonstrated that upon engagement with aberrant DNA structures arising under replication stress or during homologous recombination, the SMC5/6 complex undergoes ATPase conformational switching that transitions it from a restrained state into an activated DNA-bound architecture, enabling selective engagement of structured DNA intermediates [9,11,14]. In budding yeast, this DNA structure-dependent activation is tightly coupled to a regulatory network in which conformational changes within the Smc5–Smc6 head and coiled-coil regions relieve Nse5–Nse6–mediated inhibition, thereby licensing productive DNA association and ATP-driven clamp formation (Figure 2) [9,10,14].

This structural transition is directly coupled to localized enzymatic signaling outputs that operate at sites of DNA stress [12,16,19]. Unlike complexes that are primarily regulated by external modification, SMC5/6 contains intrinsic enzymatic modules that enable it to generate regulatory outputs directly at sites of genome instability [8,20]. The SUMO E3 ligase subunit Nse2/Mms21(NSMCE2 in mammals) is embedded within the complex and modulates the stability, localization, and interaction dynamics of multiple repair-associated proteins, thereby fine-tuning HR progression and related DNA damage responses [19,20,21]. Importantly, in budding yeast, Nse2 ligase activity is stimulated by DNA binding to a positively charged surface on the Smc5 coiled-coil arm, providing a direct mechanistic link between recognition of structured DNA and SUMOylation-dependent regulation of chromatin [16,21]. In budding yeast, SUMOylation of repair-associated factors, such as Rad52 and RPA, is consistent with a broader principle in which SUMO acts as a regulatory switch to promote productive assemblies at damage sites while restraining aberrant or excessive recombination [22,23,24]. Collectively, these findings from budding yeast and mammalian studies support a model in which SMC5/6 establishes a local SUMO-dependent control axis that biases recombination outcomes toward timely and controlled repair rather than persistent or toxic intermediates [12,25,26]. This SUMO-dependent axis represents a site-restricted signaling layer that accompanies SMC5/6 activation [12,16].

In parallel, ubiquitin signaling provides an additional layer of spatiotemporal control through the Nse1–Nse3 module [12,27]. Studies in human cell lines demonstrated that Nse1 contains a RING finger domain and, together with Nse3 (a MAGE domain-containing protein), forms a conserved E3 ubiquitin ligase that regulates complex stability, chromatin association, and dynamic retention at sites of genome stress [28]. Importantly, Nse1/Nse3-dependent ubiquitination is not primarily linked to bulk proteasomal degradation but instead modulates complex behavior. For example, in fission yeast, ubiquitination of the kleisin subunit Nse4 at lysine 181 under replication stress appears to influence residency and interaction dynamics rather than abundance, consistent with ubiquitin serving as a tuning mechanism for holocomplex integrity and damage-induced chromatin engagement [29]. In mammalian systems, SMC5/6 operates in a landscape densely regulated by ubiquitin signaling, including the Fanconi anemia pathway (e.g., FANCD2/FANCI monoubiquitination) and BRCA-associated repair circuits, suggesting a modular framework in which SMC5/6 ubiquitin regulation contributes to coordinated responses to replication-associated lesions [30,31]. Taken together, SUMO and ubiquitin signaling do not constitute independent “functions” of SMC5/6; rather, they represent regulatory layers that couple structure-specific DNA engagement to controlled execution of replication and repair programs, enabling precise responses to diverse genome instability challenges (Figure 2) [8,12].

### 3.2. SMC5/6 in Replication Stress Response and DNA Damage Repair

**Figure 3:**
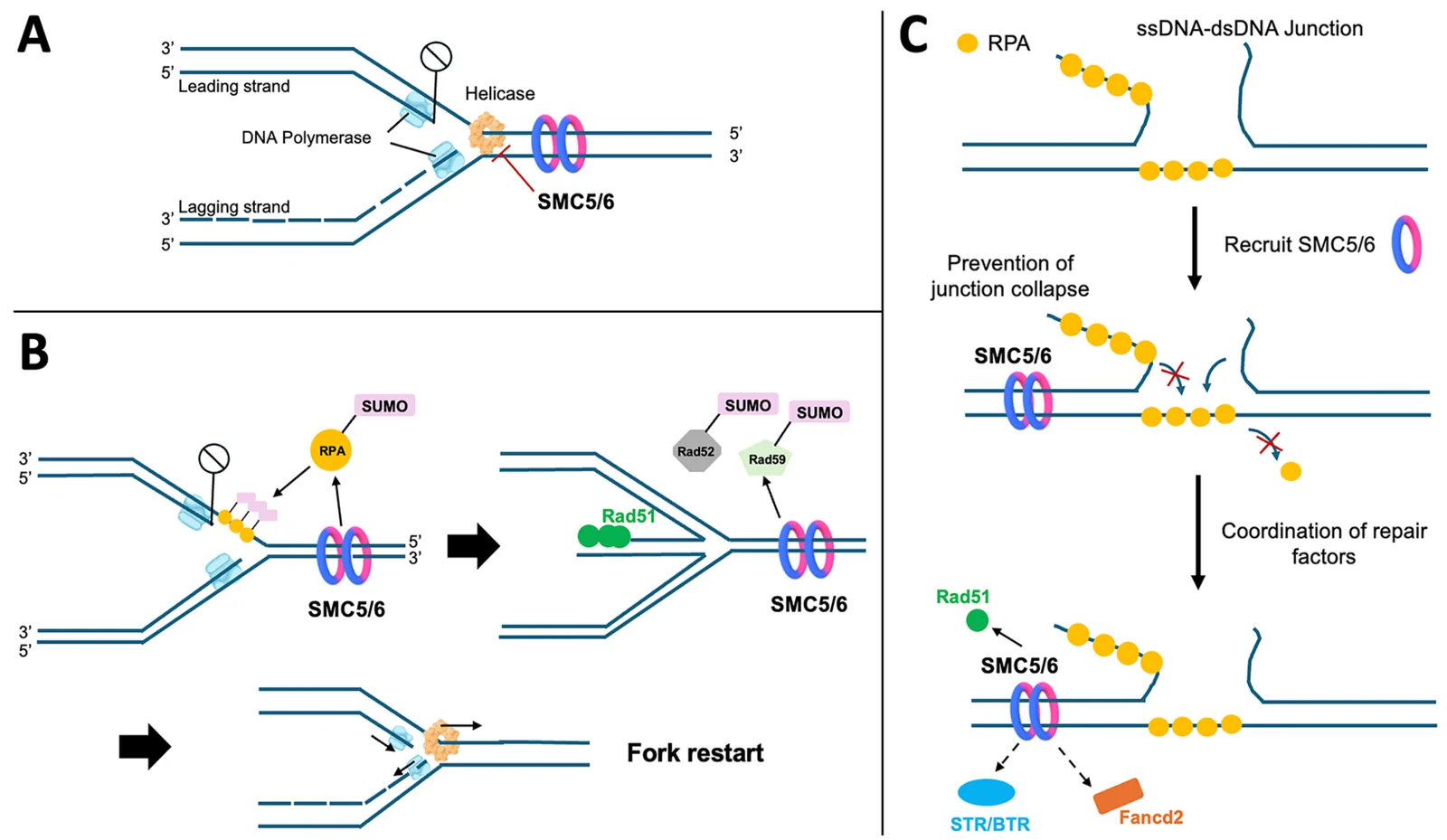
SMC5/6-mediated coordination of replication stress response and DNA damage repair. (**A**) SMC5/6 recognition and stabilization of stalled replication forks. Under replication stress, SMC5/6 associates with perturbed replication fork structures and contributes to fork stabilization by regulating fork remodeling activities. In budding yeast, Smc5/6-mediated control of the Mph1 helicase limits excessive fork regression and promotes recovery of stalled forks. (**B**) SMC5/6-dependent regulation of replication fork remodeling and restart. SUMO-dependent modification of fork-associated repair factors, including RPA and Rad52/Rad59 in yeast models, contributes to coordinated processing of replication-associated DNA structures. SMC5/6-associated homologous recombination activities, including Rad51-dependent repair processes, facilitate productive fork restart and genome stability restoration. (**C**) SMC5/6 coordination of DNA damage repair at structured DNA intermediates. SMC5/6 is recruited to ssDNA–dsDNA junctions and contributes to the regulation of repair-factor assembly. Through coordination with homologous recombination factors and repair pathways, including STR/BTR complexes and FANCD2-associated mechanisms in mammalian systems, SMC5/6 promotes controlled processing of DNA repair intermediates. The figure was created using Microsoft PowerPoint and Canva. BTR—BLM–TopoIIIα–RMI1/2 complex; FANCD2—Fanconi anemia group D2 protein; Mph1—DNA helicase Mph1; Rad51—RAD51 recombinase; Rad52—RAD52 recombination mediator; Rad59—RAD59 recombination mediator; RPA—replication protein A; SMC—Structural Maintenance of Chromosomes; SMC5/6—Structural Maintenance of Chromosomes 5/6 complex; ssDNA—single-stranded DNA; dsDNA—double-stranded DNA; STR—Sgs1–Top3–Rmi1 complex; SUMO—small ubiquitin-like modifier.

In the human system, mechanistically, SMC5/6 functions at stressed replication forks and DNA lesions as a structure-specific genome stability regulator that is functionally distinct from, yet complementary to, Cohesin [2,7]. Whereas Cohesin maintains sister chromatid cohesion and supports replication indirectly through global chromosome architecture, SMC5/6 directly recognizes and constrains stress-induced DNA topologies at the fork, thereby acting as a local sensor of replication stress-associated DNA structures [11]. This division of labor supports a model in which Cohesin provides global chromosomal organization, while SMC5/6 ensures local fork architecture integrity and suppresses pathological recombination intermediates during replication stress (Figure 3A) [2,7,8].

In the context of DNA replication, SMC5/6 is particularly important for maintaining genome stability at both rDNA and non-rDNA loci, where replication fork stalling and reversal frequently occur [8,32,33,34]. In budding yeast, SMC5/6 promotes replication fork restart at rDNA regions by restraining the helicase Mph1, thereby preventing excessive fork reversal and facilitating recovery of stalled forks [34,35,36]. In addition, in budding yeast, SMC5/6-dependent SUMO signaling contributes to the relocalization of collapsed replication forks to the nuclear pore complex, where repair is coordinated in a spatially restricted environment [24,37]. In budding yeast, this process involves SUMOylation of ssDNA- and fork-associated repair proteins, including RPA, Rad52, and Rad59, which supports productive repair-factor assembly and helps channel collapsed forks into controlled recombination-mediated repair (Figure 3B) [24,26]. SMC5 itself is also subject to SUMO modification, further suggesting that replication fork regulation is tightly coupled to post-translational control within and around the complex [16,38]. Moreover, SMC5/6 contributes to the management of replication-associated topological stress through functional cooperation with Topoisomerase II, thereby ensuring smooth progression of the replication machinery across difficult genomic regions [12,39,40].

Genetic studies in budding yeast and human cell systems have demonstrated that SMC5/6 plays a central role in homologous recombination (HR)–mediated DNA damage repair by controlling the formation, stability, and resolution of recombination intermediates. In both budding yeast and human cells, SMC5/6 promotes sister chromatid-based HR repair and helps suppress inappropriate recombination events that could otherwise compromise genome integrity [41,42]. At DNA lesions that generate ssDNA–dsDNA junctions, RPA-coated single-stranded DNA and branched repair intermediates provide repair-associated platforms for SMC5/6 function. Once engaged, SMC5/6 helps stabilize vulnerable DNA junctions, supports productive RPA/Rad52-associated repair intermediates, and prevents the persistence or collapse of aberrant joint molecules [32,43]. This local stabilization is closely coupled to SUMO-dependent repair-factor coordination. In budding yeast, SMC5/6-mediated SUMO signaling promotes the regulation of Rad51-dependent recombination factors and the Sgs1–Top3–Rmi1 (STR) complex, thereby facilitating the timely processing of HR intermediates and favoring non-crossover repair outcomes; related functions are also linked to BLM–TOP3A–RMI1/2 and FANCD2-associated repair signaling in metazoan cells (Figure 3C) [25,44,45]. Consistent with these functions, genetic disruption of SMC5/6 in yeast and mammalian cells leads to excessive HR intermediate accumulation, increased sister chromatid exchange, and elevated gross chromosomal rearrangements, highlighting its central role in suppressing aberrant recombination during DSB repair [46,47,48]. SMC5/6 can also modulate HR indirectly through cohesin regulation and cohesin SUMOylation, thereby influencing sister chromatid-based recombination outcomes [42,49]. Beyond canonical HR repair, in Drosophila cells, SMC5/6 participates in heterochromatin DNA repair by restraining unscheduled recombination within heterochromatin and facilitating actin–myosin-dependent relocalization of DNA lesions to more permissive nuclear compartments. Furthermore, SMC5/6 has been implicated in alternative lengthening of telomeres (ALT), where it supports ALT-associated PML body formation and telomere maintenance through SUMOylation of telomere-binding proteins, reinforcing its broader role in genome stability across diverse chromatin contexts [50,51,52].

In budding yeast, recruitment of SMC5/6 to DNA damage sites is mediated by context-dependent pathways. During G2/M, SMC5/6 is recruited to DNA double-strand breaks through early damage-sensing mechanisms involving the MRX complex, whereas additional recruitment routes operate during replication stress and collapsed-fork repair [53]. Another recruitment route depends on cooperation between the Nse5–Nse6 subcomplex and BRCT-domain scaffold proteins. In budding yeast, Rtt107 promotes SMC5/6 recruitment to DNA double-strand breaks, while in fission yeast, Brc1 supports Smc5–Smc6 focal accumulation and SUMO ligase activity during replication stress [54,55]. In vertebrate systems, SMC5/6 localization to damaged or replication-stressed chromatin relies in part on the RAD18–SLF1–SLF2 adaptor pathway. SLF1–SLF2 links RAD18-dependent DNA damage signaling to SMC5/6 recruitment, and recent structural work further explains how SLF1 recognizes RAD18 and chromatin-associated features at stalled replication forks [56,57]. Together, these findings support a conserved recruitment logic in which species-specific adaptor modules target SMC5/6 to damaged or replication-stressed chromatin enriched in structured DNA intermediates [12,58].

## 4. SMC5/6-Mediated Antiviral Restriction and Viral Evasion Mechanisms

The SMC5/6 complex, known for its fundamental role in maintaining genome stability, has emerged as a critical antiviral factor that specifically targets episomal viral DNA [8]. Several restriction mechanisms have been identified, including episome silencing through chromatin compaction and SUMOylation for human immunodeficiency virus 1 (HIV-1) and Kaposi’s sarcoma-associated herpesvirus (KSHV) [59,60], and recruitment to nuclear domains such as ND10 for hepatitis B virus (HBV) silencing [61,62]. Additionally, direct competition represents another mechanism, as observed in human papillomavirus (HPV), where SMC5/6 competes with viral E2 protein for binding to the E1 replication protein [63]. Other mechanisms have been proposed; however, they remain to be comprehensively studied.

**Figure 4:**
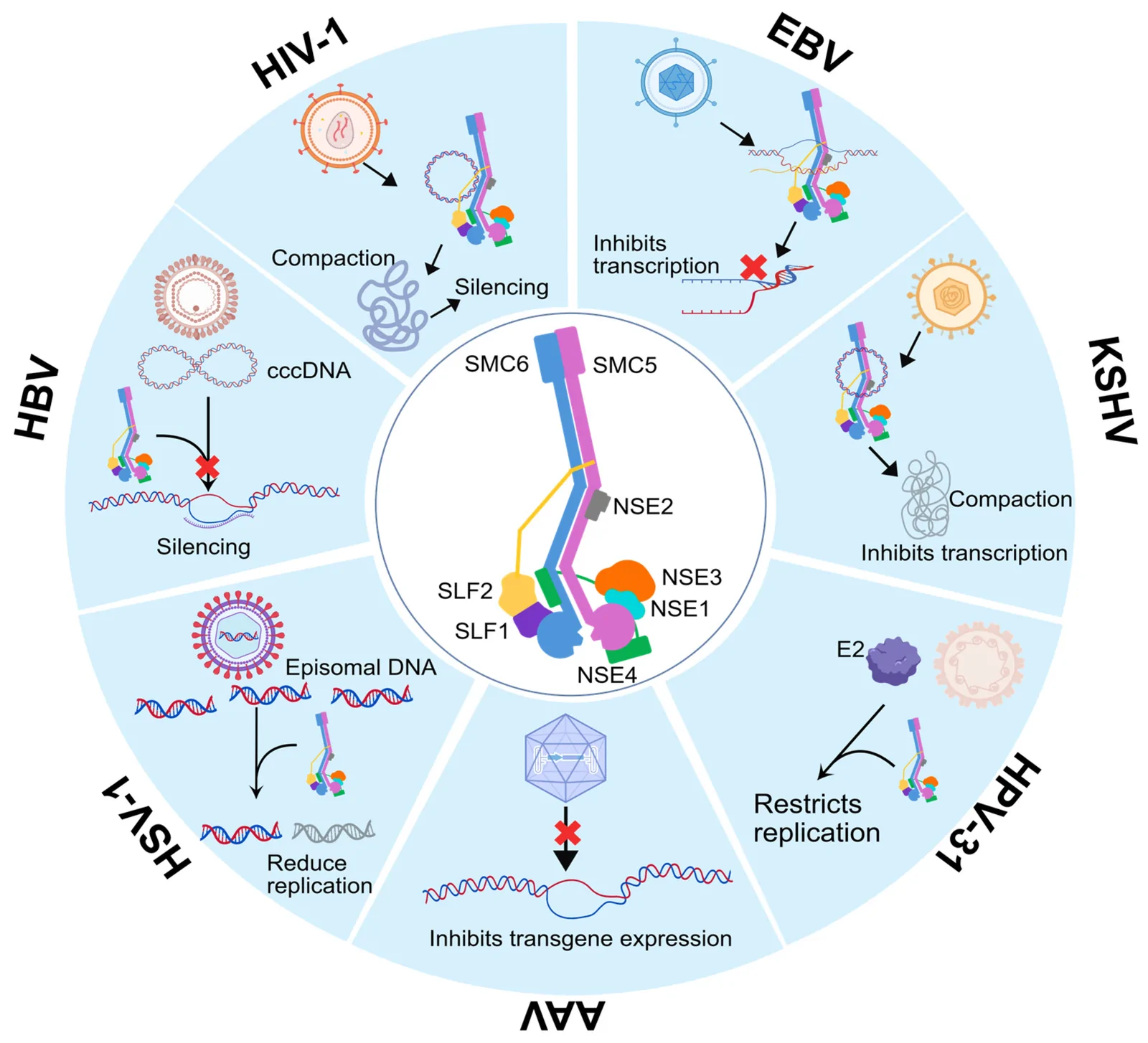
SMC5/6 Complex-Mediated Restriction of Viruses. Created with BioGDP.com [72].

The molecular details of SMC5/6-mediated restriction have been particularly well-characterized for HBV. HBV regulatory protein X (HBx) activates gene expression from the HBV covalently closed circular DNA (cccDNA) genome. It was known that HBx bound the DNA-damage binding protein 1 (DDB1) subunit of a DDB1-containing E3 ubiquitin ligase. Using a proteomic approach, 2 groups identified the SMC5/6 complex as a target for binding by the HBx-DDB1 E3 ligase complex and showed that this induced the degradation of SMC5 and SMC6. Moreover, the SMC5/6 complex directly bound episomal HBV DNA in the absence of HBx, and loss of SMC5/6 complex function rescued the replication of an HBV mutant lacking a functional HBx [61,62]. SMC5/6 knockdown, or inhibition with a dominant-negative SMC6, enhances HBx null mcHBV-Gluc gene expression and rescues HBx-deficient HBV replication in human hepatocytes [62]. Together, these data indicated that the SMC5/6 complex recognizes and binds episomal HBV DNA molecules and then induces their epigenetic silencing. HBx, in turn, prevents this silencing by inducing the degradation of SMC5 and SMC6. Thus, it is only in the context of infection by an HBx-deficient HBV mutant that the antiviral activity of the SMC5/6 complex is revealed. Research also suggests that SMC5/6 suppresses HBV transcription when localized to ND10 without inducing a detectable innate immune response [64]. Recent research has suggested that the SMC5/6 complex restricts HBV by a three-step process [12]. The first step is entrapment of the episomal DNA by a mechanism dependent on SMC5/6 ATPase activity and a function of its Nse4a subunit, for which the Nse4b paralog cannot substitute. The second step results in SMC5/6 recruitment to promyelocytic leukemia nuclear bodies by SLF2 (the human ortholog of Nse6). The third step promotes silencing through a mechanism requiring Nse2 but not its SUMO ligase activity (Figure 4) [65]. The recruitment step depends on SIMC1, a paralog of SLF1 that forms an NSE5/NSE6-like complex with SLF2 and behaves similarly to its yeast counterpart in the localization of the SMC5/6 complex to specific regions of chromosomes [58]. Recently, researchers have provided structural insights into this process by resolving the HBx–CRL4–Smc5/6 complex, revealing that HBx employs a Zn^2+^-stabilised helix-turn-helix pocket to engage a conserved leucine key motif on Smc6, thereby recruiting CRL4 to ubiquitinate and degrade the SMC5/6 complex [66]. This study also demonstrated that the small molecule Tranilast disrupts this interface and restores SMC5/6-mediated antiviral silencing, offering a potential therapeutic strategy for HBV.

For HIV-1, the restriction landscape differs significantly due to the essential integration step in retroviral replication. A key difference between HBV and retroviruses such as HIV-1 is that retroviruses include chromosomal integration of the DNA proviral intermediate as an essential step in their replication cycle. In the absence of integrase (IN) function, unintegrated linear HIV-1 DNA generated by reverse transcription of the viral RNA genome is rapidly chromatinized and epigenetically silenced [67]. Notably, the HIV-1 accessory protein Vpr is known to enhance the expression of unintegrated viral DNA, suggesting it antagonizes a cellular restriction factor. Through a targeted CRISPR-Cas9 screen, the researchers identified SLF2 as the specific Vpr target responsible for this silencing. SLF2 functions as a recruitment factor that directs the SMC5/6 complex to unintegrated lentiviral DNA. Depleting either SLF2 or the SMC5/6 complex itself increases viral gene expression. The mechanism of silencing was further revealed by ATAC-seq analysis, which showed that Vpr-mediated depletion of SLF2 increases the chromatin accessibility of the unintegrated virus. This indicates that the SMC5/6 complex functions to compact viral chromatin, thereby suppressing gene expression (Figure 4) [59]. A second CRISPR/Cas genomic screen reported that all 8 components of the SMC5/6 complex, including SLF1, were required for effective silencing. This study also identified the SUMO E3 ligase activity of the SMC5/6 complex component NSMCE2 as essential for silencing. Specifically, a mutant form of NSMCE2 lacking SUMO E3 ligase activity remained able to bind chromatinized, unintegrated HIV-1 DNA, yet had lost the ability to induce both its SUMOylation and its epigenetic silencing. Moreover, a drug that acts as a general inhibitor of protein SUMOylation, called TAK-981, also rescued gene expression from unintegrated HIV-1 proviruses. SUMOylation by the NSMCE2 component of SMC5/6 was required for the initiation but not maintenance of epigenetic silencing, as the ability of TAK-981 to prevent silencing was lost after 24 h post-infection, even though TAK-981 retained the ability to deplete SUMO modifications from chromatinized, unintegrated HIV-1 DNA at later times [68].

Furthermore, the restrictive function of SMC5/6 extends across a diverse range of DNA virus families. Epstein–Barr virus employs its BNRF1 tegument protein to induce SMC5/6 ubiquitination and degradation. When this degradation is blocked, SMC5/6 binds to episomal Epstein–Barr virus (EBV) DNA, disrupts replication compartment formation, and inhibits virion production (Figure 4). This restriction involves the SUMOylation activity of the NSMCE2 (NSE2) subunit [69]. For KSHV, the SMC5/6 complex binds to episomal KSHV DNA to inhibit replication (Figure 4). The SMC5/6 complex can bind to the KSHV genome and suppress KSHV gene transcription by condensing the viral chromatin and creating a repressive chromatin structure. KSHV counteracts this via its RTA protein, which ubiquitinates and degrades SMC5/6 components [60]. As for HPV, the SMC5/6 complex binds to the HPV-31 E2 protein, and its depletion increases viral replication in keratinocytes, indicating a restrictive role, though the mechanistic basis for this effect remains unknown (Figure 4) [63]. Similarly, it has been reported that herpes simplex virus 1 (HSV-1) replication is reduced through SMC5/6 binding to episomal viral DNA (Figure 4) [70]. The complex also inhibits transgene expression from adeno-associated virus (AAV) vectors, and SUMOylation has been implicated in this restriction (Figure 4) [71]. The broad targeting of SMC5/6 by diverse viruses underscores its fundamental role as a cellular sentinel against foreign DNA, with viral antagonists consistently evolving to dismantle this defense system through degradation (Table 1).

**Table 1:** Reported roles of SMC5/6-Mediated Viral Restriction and Viral Antagonism.

Viruses	SMC5/6 Restriction Mechanism	Viral Antagonism Mechanism
HBV [4,61,62,73]	ATPase- and Nse4a-dependent capture of HBV episomal cccDNA, SLF2/SIMC1-mediated recruitment to PML nuclear bodies, and SUMO ligase-independent cccDNA silencing via Nse2.	HBx protein hijacks the CRL4-DDB1/E3 ubiquitin ligase to target SMC5/SMC6 subunits for ubiquitination and degradation.
HIV-1 [59,68]	SLF2 recruits SMC5/6 to unintegrated viral DNA, leading to chromatin compaction and transcriptional silencing. SUMOylation by NSMCE2 is essential for initiating silencing.	Vpr protein hijacks the CRL4-DCAF1/ E3 ubiquitin ligase to target SLF2 for degradation, preventing SMC5/6 recruitment.
EBV [69]	Binds episomal DNA and inhibits replication, a process involving the SUMOylation activity of NSMCE2.	BNRF1 tegument protein induces the ubiquitination and degradation of the SMC5/6 complex.
KSHV [60]	Binds the viral episome and suppresses transcription by condensing viral chromatin.	RTA protein induces the ubiquitination and degradation of SMC5/6 components.
HPV-31 [63]	Binds the viral E2 protein and likely restricts replication through direct competition with E2 for the E1 helicase.	The mechanism of evasion is not fully known, but SMC5/6 depletion enhances viral replication.
HSV-1 [70]	Binds episomal viral DNA and reduces viral replication (precise mechanism less defined).	Not specified.
AAV [71]	Inhibits transgene expression from vectors, with SUMOylation implicated in the restriction.	Not specified.

## 5. The Critical Roles of the SMC5/6 Complex in Hereditary Developmental Disorders

SMC5/6 plays an important role in homologous recombination repair, which can promote the accurate repair of broken DNA strands and prevent the accumulation of mutations caused by non-homologous end joining [22,41,42]. When the SMC5/6 function is impaired, cells will undergo replication fork arrest, unrepaired DNA double-strand breaks, and chromosomal mismatching and breakage, leading to genomic instability [74,75].

Studies have shown that mutations in the SMC5/6 complex are closely associated with various hereditary developmental disorders in human patients [5,48,74]. Among the disease-associated subunits, NSMCE2 (also known as NSE2 or MMS21), the SUMO E3 ligase component of the SMC5/6 complex, has attracted considerable attention [5]. NSE2-mediated SUMOylation is essential for regulating the recruitment and activity of proteins involved in DNA repair and replication stress responses, as validated in human cellular models [22,42,76]. Studies have demonstrated that severe deficiency of human NSE2 or mutations that abolish its SUMO ligase activity compromise the structural integrity and stability of the entire SMC5/6 complex [77,78]. Consequently, human patient-derived affected cells exhibit increased chromosomal aberrations, replication fork instability, accumulation of DNA damage, and defective homologous recombination repair [42,74]. Clinically, patients carrying pathogenic NSMCE2 variants develop a multisystem developmental syndrome characterized by primordial dwarfism, microcephaly, developmental delay, and metabolic and endocrine abnormalities [5,8]. The broad spectrum of phenotypes suggests that impaired SMC5/6 function disrupts cellular proliferation and tissue homeostasis across multiple human organ systems [5,74]. Notably, the presence of growth retardation and microcephaly resembles phenotypes observed in other genome instability syndromes, supporting the concept that defective DNA maintenance pathways are major contributors to developmental disorders [8].

Another important example involves NSMCE3 (NSE3), a core non-SMC component that contributes to DNA binding and the structural organization of the SMC5/6 complex [79]. Biallelic NSMCE3 mutations have been identified in patients presenting with severe adaptive immunodeficiency accompanied by rapidly progressive pulmonary disease [80]. Functional analyses revealed that these mutations impair SMC5/6-mediated genome maintenance, leading to increased sensitivity to DNA damage and defective lymphocyte development [8,80]. Because adaptive immune cells undergo extensive proliferation, DNA replication, and programmed genomic rearrangements during maturation in human individuals, they are particularly vulnerable to deficiencies in chromosome maintenance pathways [81,82]. As a result, affected individuals experience profound immune dysfunction and recurrent respiratory complications, often leading to premature death during childhood [80]. These findings further demonstrate that SMC5/6 activity is essential not only for general developmental processes but also for the maintenance of specialized cell populations with high proliferative demands.

More recently, mutations in SMC5 itself and in SLF2, a critical factor that recruits the SMC5/6 complex to sites of DNA damage in human cells, have been shown to cause a newly recognized chromosome instability disorder known as Atelís syndrome (ATS) [48]. Patients carrying biallelic SMC5 or SLF2 mutations display severe microcephaly, proportionate short stature, developmental delay, and learning disabilities, indicating a fundamental requirement for the SLF2–SMC5/6 pathway during human growth and brain development [48]. In addition to neurodevelopmental abnormalities, affected individuals frequently exhibit congenital heart defects, hematological abnormalities, and, in some cases, myelodysplastic syndrome [48]. Cellular analyses of patient-derived human cells revealed elevated replication stress, chromosome breakage, abnormal sister chromatid organization, and a characteristic phenotype of segmented chromosomes and mosaic variegated hyperploidy [48,81]. Experimental studies using zebrafish models further demonstrated that loss of slf2 or smc5 leads to impaired neural progenitor proliferation, increased apoptosis, and defective craniofacial development [48]. These zebrafish-based findings indicate an evolutionarily conserved role of the SLF2–SMC5/6 axis in embryonic genome stability maintenance. However, phenotypic abnormalities observed in zebrafish models are relatively simple and limited to developmental morphological defects, whereas human patients exhibit more complex, multisystem clinical manifestations and unique chromosomal aberrations that are not fully recapitulated in zebrafish. Therefore, these model-specific phenotypes cannot be generalized to human developmental processes. Currently reported SMC5/6 complex subunits involved in hereditary developmental disorders, as well as their protein functions, clinical abnormal phenotypes, and molecular pathogenic mechanisms, are summarized in Table 2.

## 6. Mutation Spectrum Analysis of SMC5/6 Complex Genes in Human Cancers

Maintenance of genome integrity is essential for the normal proliferation and survival of eukaryotic cells. Defects in DNA damage repair and chromosome homeostasis contribute to genomic instability and are closely associated with tumor initiation and progression [83,84,85,86]. As a key regulator of chromosome maintenance, the SMC5/6 complex plays critical roles in DNA replication, DNA repair, and chromosome segregation. Recent large-scale cancer genomic studies have revealed widespread genetic alterations in genes encoding SMC5/6 complex subunits across multiple human malignancies, including missense mutations, truncating mutations, and copy number alterations. Functional studies have shown that certain cancer-associated mutations impair cellular responses to DNA damage and replication stress, leading to compromised genome stability and suggesting that SMC5/6 mutations may represent an important molecular mechanism underlying complex dysfunction and cancer development [6,87,88,89].

To investigate the mutational characteristics and potential clinical significance of the nine subunit genes of the SMC5/6 complex (SMC5, SMC6, SLF2, SIMC1, SLF1, NSMCE1, NSMCE2, NSMCE3, and NSMCE4A) in human tumors, this study was performed based on large-scale data from the Pan-Cancer Atlas initiative of The Cancer Genome Atlas (TCGA). Somatic mutation data were obtained from the cBioPortal cancer genomics database (https://www.cbioportal.org/; accessed 19 January 2026), with the query restricted to the 32 TCGA PanCancer Atlas studies, which provide uniformly reprocessed somatic mutation calls derived from the TCGA MC3 project; germline variants are excluded upstream by this pipeline, and no additional filtering on allele frequency or sequencing depth was applied. Mutation-level records—including gene symbol, study of origin, cancer type, protein change (HGVS p. notation), mutation type, genomic coordinates, and functional impact annotations (MutationAssessor, SIFT, PolyPhen-2, and AlphaMissense)—were exported from the Mutations tab of the query results, yielding a total of 997 somatic mutation records for the nine subunit genes of the SMC5/6 complex across 26 TCGA cohorts, which cBioPortal aggregates into 23 cancer type categories according to the OncoTree classification. All somatic mutation classes reported by cBioPortal were retained, comprising missense (*n* = 784, 78.6%), nonsense (*n* = 100), frameshift (*n* = 53), splice-site/splice-region (*n* = 36), gene fusion (*n* = 11), in-frame insertion/deletion (*n* = 8), and other rare classes (*n* = 5). These records were compiled for subsequent analyses, all of which were performed in Python 3.12.2 with pandas 2.2.3, NumPy 1.26.4, Matplotlib 3.9.2, and seaborn 0.13.2; the compiled mutation table and analysis scripts are available from the corresponding author upon reasonable request. This study aims to reveal the mutational patterns of the SMC5/6 complex across pan-cancer, so as to provide fundamental data and theoretical references for further understanding its molecular mechanisms in tumorigenesis and exploring potential targets for cancer diagnosis and treatment.

**Figure 5:**
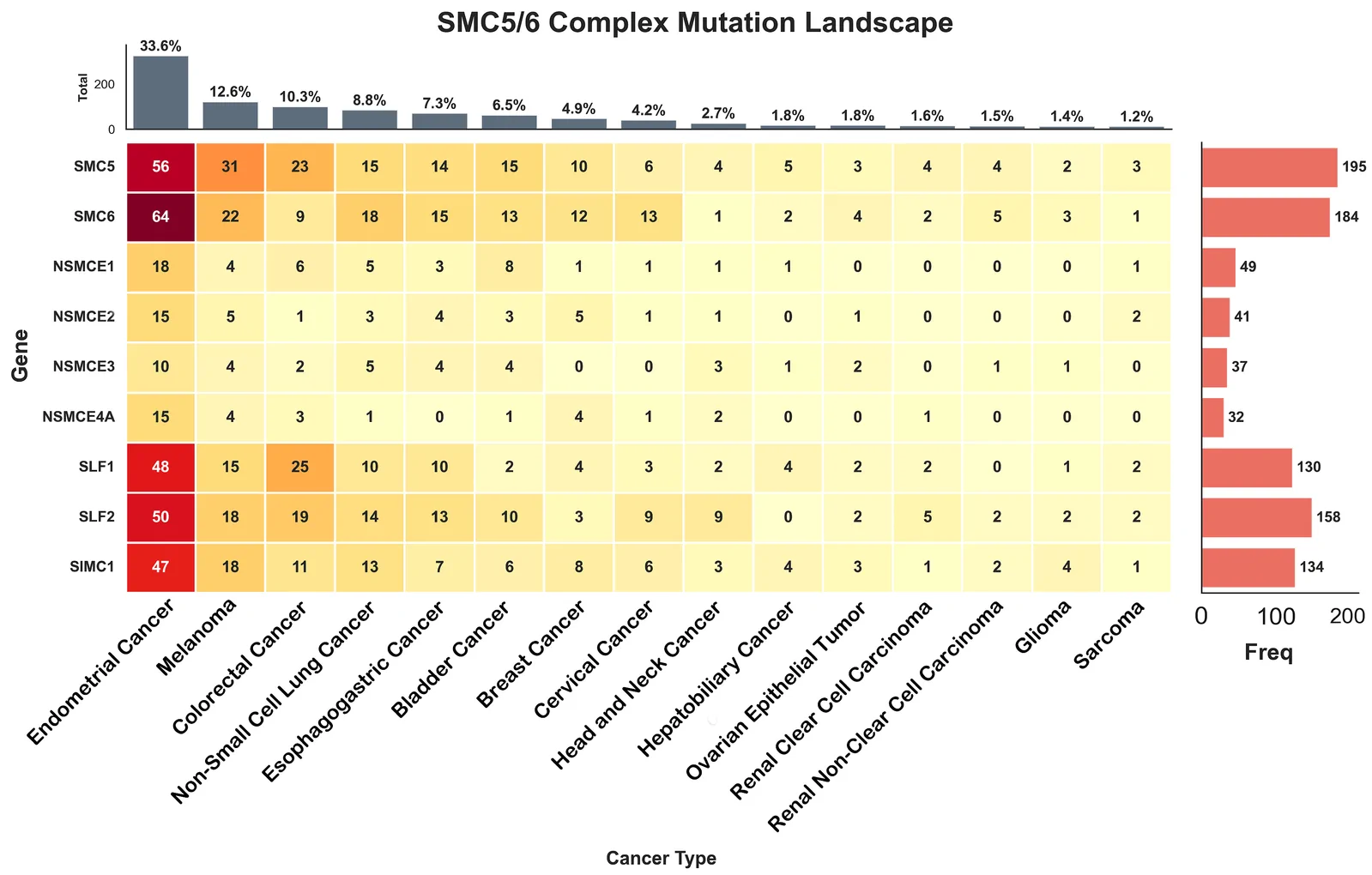
Mutation Landscape of SMC5/6 Complex Subunits Across 15 Human Cancer Types. Mutation data of SMC5/6 complex subunits were retrieved from cBioPortal for Cancer Genomics (https://www.cbioportal.org/) accessed on 19 January 2026; the query was restricted to the 32 TCGA PanCancer Atlas studies, and the 15 cancer types with the largest numbers of mutation records are shown (960/997 records, 96.3%). Data preprocessing was performed using Python 3.12.2 with pandas 2.2.3 and NumPy 1.26.4, and visualization was implemented with Matplotlib 3.9.2 and seaborn 0.13.2. Each heatmap cell reports the number of mutation records for the corresponding gene × cancer type pair. The top marginal bar shows the total number of records per cancer type, annotated with its percentage of all records among the 15 cancer types (P(c) = N(c)/960 × 100%), and the right marginal bar shows the total number of records per gene. In the heatmap, the color intensity is positively correlated with the mutation count: darker colors (such as red and orange) indicate higher mutation counts, while lighter colors (such as light yellow) represent lower mutation counts, which intuitively reflects the differences in mutation levels of SMC5/6 complex subunits among different cancer types.

Based on the mutation data of each subunit of the SMC5/6 complex obtained from the cancer genomics cBioPortal platform, this study systematically analyzed the mutation characteristics and distribution patterns of each subunit of the complex in the 15 human cancer types harboring the largest numbers of mutation records (960/997 records, 96.3%; each of the eight excluded cancer types contributed ≤ 10 records). Unless otherwise stated, the percentages reported below denote each cancer type’s share of all mutation records among these 15 cancer types, P(c) = N(c)/ΣN(c′) × 100% (ΣN(c′) = 960), rather than per-sample alteration frequencies within each cohort. As shown in the mutation heatmap, the distribution of SMC5/6 complex mutation records varied significantly among different tumor types, with endometrial cancer accounting for the largest proportion of mutation records (33.6%, 323/960), followed by melanoma (12.6%, 121/960), colorectal cancer (10.3%, 99/960), and non-small cell lung cancer (8.8%, 84/960). Among the core subunits of the SMC5/6 complex, SMC5 and SMC6 had the highest total mutation counts within the 15 visualized cancer types, 195 and 184, respectively, and the mutations of these two subunits were particularly enriched in endometrial cancer, with 56 and 64 mutations, respectively. In addition to the core subunits, other components of the complex (SLF1, SLF2, SIMC1) also exhibited relatively high mutation counts, among which SLF1 had a total of 130 mutations, SLF2 158, and SIMC1 134. The mutation counts of these three subunits in endometrial cancer were 48, 50, and 47, respectively, which further confirmed the marked enrichment of SMC5/6 complex mutations in endometrial cancer. In contrast, cancers such as glioma and sarcoma each accounted for a minimal fraction of SMC5/6 complex mutation records (<2%; 13/960 and 12/960, respectively) (Figure 5). The analysis results indicated that the SMC5/6 complex harbored mutations in some human cancers, and this mutation characteristic was most prominent in endometrial cancer. This suggests that dysfunction of the SMC5/6 complex, which serves as the core of DNA damage repair and genomic stability regulation, may be a key driving event in the occurrence and development of such tumors, providing an important basis for further exploration of the role of this complex in tumor pathogenesis and its potential clinical application value.

**Figure 6:**
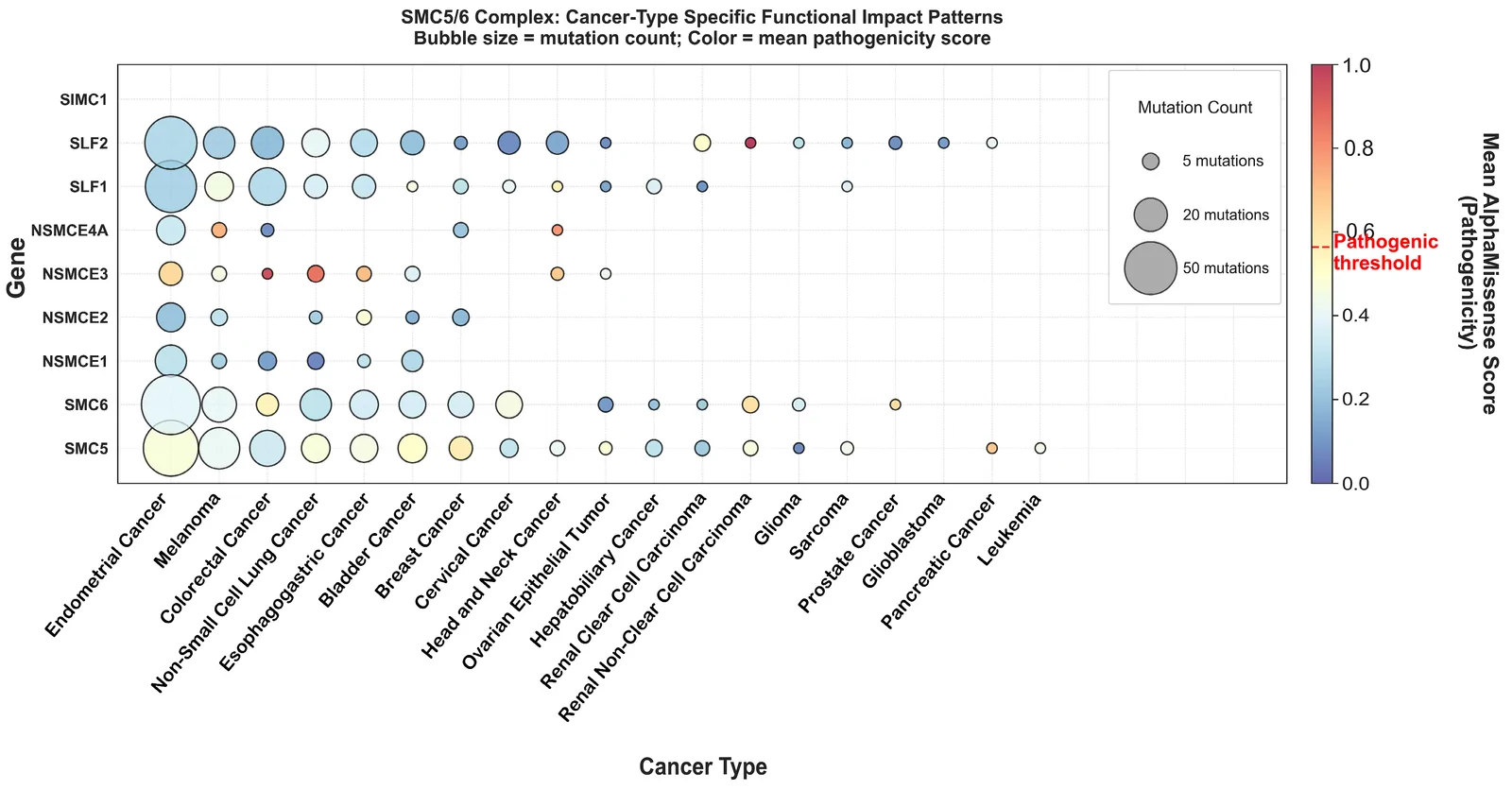
This bubble plot illustrates the mutational landscape and functional impact of missense mutations in SMC5/6 complex subunits across diverse cancer types. Only gene × cancer type pairs with at least two mutation records and at least one AlphaMissense-scored mutation are shown. Each bubble represents a distinct cancer type–gene pair (i.e., one gene combined with one cancer type): bubble size corresponds to the total number of missense mutations in the pair, with reference scales of 5, 20, and 50 mutations provided in the legend. Bubble color encodes the mean AlphaMissense score of all scored missense mutations within the pair (ranging from 0 to 1), with a gradient from blue (low pathogenicity, scores approaching 0) to red (high pathogenicity, scores approaching 1). The red dashed line adjacent to the color bar indicates the pathogenicity threshold (AlphaMissense score ≥ 0.564), above which mutations are classified as pathogenic. AlphaMissense score data were derived from cBioPortal for Cancer Genomics.

We further analyzed the mutation distribution and potential functional impacts of genes related to the SMC5/6 complex across different cancer types. For these analyses, AlphaMissense pathogenicity scores were parsed from the cBioPortal functional-impact annotation and were available for 667 of the 784 missense mutations (85.1%); non-missense and unscored variants were excluded from all score-based summaries, and mutations with a score ≥ 0.564 were considered likely pathogenic (165/667, 24.7%). Consistent with the previous mutation landscape analysis, endometrial cancer had the highest mutation count among core subunits, including SMC5, SMC6, SLF1, SLF2, and SIMC1, highlighting its high mutational burden. Melanoma, colorectal cancer, and non-small cell lung cancer also had significant mutations in key subunits, suggesting that the complex may be involved in the occurrence of these tumors. In contrast, glioma, sarcoma, and leukemia showed extremely low mutation frequencies in all subunits, indicating a weak correlation between the complex and these tumor types. Notably, the mean AlphaMissense scores of each subunit varied significantly among different cancers and subunits, with obvious specificity. In non-small cell lung cancer, non-core subunits such as NSMCE3 and NSMCE4A had persistent pathogenicity scores > 0.6, and some were close to 0.8, suggesting that these mutations are functional and may directly drive tumorigenesis by disrupting protein function. Although endometrial cancer had the highest mutation count, the pathogenicity scores of most subunits were concentrated between 0.2 and 0.5, with only a few subunits such as SIMC1 having individual high-pathogenicity mutations. This indicates obvious heterogeneity in the mutations of the SMC5/6 complex in endometrial cancer, including both driver mutations that may promote tumor occurrence and a large number of passenger mutations that do not affect tumor progression, with a higher proportion of passenger mutations. In addition, the pathogenicity scores of all subunits of the SMC5/6 complex in cancers such as breast cancer and bladder cancer were generally between 0.3 and 0.6 (Figure 6), without obvious concentration tendency, suggesting that the functional impact of these mutations is mild and may only play an auxiliary regulatory role in specific tumor microenvironments or genetic backgrounds. These results indicate that the functional impacts of SMC5/6 complex mutations have clear cancer-type and subunit specificity, revealing the mutation characteristics of different cancers and the pathogenic heterogeneity of different subunits. This provides an accurate experimental basis for clarifying its mechanism in tumorigenesis, screening potential targets, and conducting precise treatment research.

**Figure 7:**
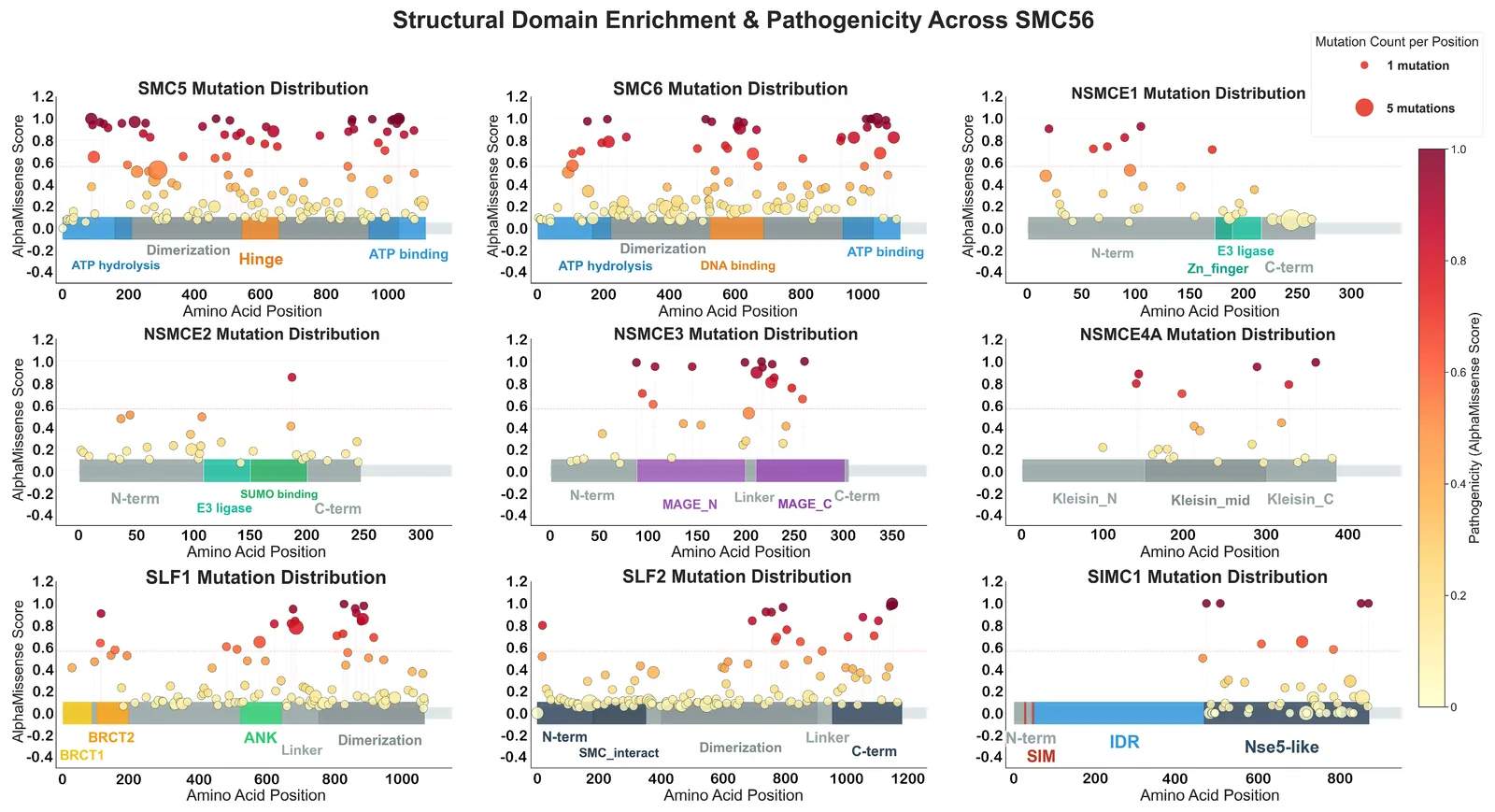
Structural Domain Enrichment and Pathogenicity Across SMC5/6 Complex Subunits. Protein domain boundaries were manually curated from the corresponding UniProt entries (https://www.uniprot.org; distributed under the CC BY 4.0 license; SMC5, Q8IY18; SMC6, Q96SB8; NSMCE1, Q8WV22; NSMCE2, Q96MF7; NSMCE3, Q96MG7; NSMCE4A, Q9NXX6; SLF1, Q9BQ83; SLF2, Q8IX21; SIMC1, Q8NDZ2) and published structural studies of the SMC5/6 complex. These lollipop plots visualize the distribution of mutations across the amino acid sequences of SMC5/6 complex subunits. Amino acid positions were extracted from the HGVS protein-change annotation, and mutation records were aggregated per residue. Each dot represents a single mutation site: Dot size corresponds to the mutation count at each site and reflects the frequency of mutation occurrence. Dot color encodes the AlphaMissense pathogenicity score, with a gradient from yellow (low pathogenicity, scores near 0) to red (high pathogenicity, scores near 1), where scores ≥0.564 are classified as pathogenic.

We analyzed the amino acid-level mutational distribution, structural domain enrichment patterns, and pathogenicity differences across all subunits of the SMC5/6 complex, providing a high-resolution perspective for understanding the mechanisms underlying functional dysregulation of this complex in tumors. For this analysis, amino acid positions were extracted from the HGVS protein-change notation (records without a parseable position, e.g., gene fusions, were excluded) and mutation records were aggregated per residue, with protein domain boundaries manually curated from the corresponding UniProt entries supplemented by published structural studies of the SMC5/6 complex. Mutations are broadly distributed across core structural subunits SMC5 and SMC6, with prominent enrichment in functional domains including the ATP hydrolysis, hinge/DNA binding, and ATP binding regions, consistent with their essential roles in maintaining genome stability. For regulatory subunits, mutations are concentrated in key functional modules: NSMCE1/2 mutations cluster in E3 ligase and SUMO-binding domains, NSMCE3 mutations are enriched in MAGE_N/MAGE_C domains, and NSMCE4A mutations localize to Kelch domains, suggesting that SUMOylation, protein stability, and substrate-binding functions are recurrently targeted in tumorigenesis. Among accessory subunits, SLF1 mutations accumulate in BRCT and ANK domains involved in DNA damage response, while SLF2 mutations are enriched in SMC-interacting and dimerization domains that mediate complex assembly. High-pathogenicity mutations (marked by red bubbles with high AlphaMissense scores) are not randomly distributed but preferentially localize to these core functional domains, indicating that tumor-derived mutations selectively disrupt the catalytic, DNA-binding, and protein-interaction interfaces of the SMC5/6 complex. Notably, while some regions exhibit high mutation counts (large bubbles), many high-pathogenicity mutations occur as discrete, low-frequency hotspot residues, implying that these single-site alterations may act as key drivers of oncogenic dysfunction (Figure 7). Collectively, these data reveal a domain-targeted pattern of mutation and pathogenicity in the SMC5/6 complex.

## 7. Conclusions and Perspectives

Recent advances in structural biology, genetics, and cell biology have substantially expanded our understanding of the SMC5/6 complex. Accumulating evidence indicates that SMC5/6 plays essential roles in maintaining genome stability through its involvement in DNA replication, homologous recombination, chromosome segregation, and the cellular response to replication stress [33,43,74,75,90,91,92]. In addition to these established functions, recent studies have revealed unexpected roles for SMC5/6 in transcriptional regulation and antiviral restriction [4,65], highlighting the functional diversity of this complex across different biological contexts. These findings have broadened the current view of SMC5/6 from a DNA repair-associated factor to a multifunctional regulator of genome maintenance and chromatin-associated processes.

Recent cryo-electron microscopy studies have provided important insights into the architecture and conformational dynamics of the SMC5/6 complex. Structural analyses have revealed several distinct conformational states and have identified regulatory subunits that contribute to complex assembly and activity [10,17,93,94,95]. Nevertheless, many mechanistic questions remain unresolved. In particular, how ATP hydrolysis is coordinated with DNA binding and release, how different subunits cooperate during replication stress and DNA repair, and how structural changes are linked to downstream cellular functions remain incompletely understood. Addressing these questions will be essential for establishing a comprehensive mechanistic framework for SMC5/6 function in genome maintenance.

In addition to its established functions in genome maintenance, increasing evidence has identified the SMC5/6 complex as an important antiviral restriction factor. Studies in recent years have demonstrated that SMC5/6 can suppress the transcription and persistence of episomal viral genomes, and that multiple unrelated viruses have evolved mechanisms to counteract or evade SMC5/6-mediated restriction [4,60,63,65,66,67,68,69,70,71]. These findings highlight the broader biological significance of SMC5/6 beyond chromosome maintenance and suggest an intimate connection between genome surveillance pathways and host antiviral defense. Despite these advances, the molecular mechanisms underlying SMC5/6-mediated antiviral activity remain incompletely understood. In particular, how SMC5/6 is recruited to viral episomes, how it establishes transcriptional repression, and how viral proteins antagonize these processes require further investigation. Addressing these questions will not only improve our understanding of SMC5/6 biology but may also provide new opportunities for the development of antiviral therapeutic strategies targeting virus–host interactions.

The growing association between SMC5/6 dysfunction and human disease further underscores the biological importance of this complex. Developmental disorders caused by inherited mutations in SMC5/6-associated genes have demonstrated their essential role in normal development and genome maintenance [5,48,80]. In addition, recent cancer genomic studies have revealed recurrent genetic alterations affecting multiple components of the SMC5/6 complex across diverse tumor types, suggesting a broader involvement in human disease [6,89]. However, the functional consequences of many disease-associated variants remain poorly understood. In particular, it remains unclear whether these alterations directly contribute to tumor development and progression or simply reflect the increased mutational burden characteristic of genomically unstable cancers. Future studies integrating structural biology, functional genomics, cellular models, and in vivo disease systems will be necessary to establish genotype–phenotype relationships and clarify the potential clinical significance of SMC5/6-associated alterations.

Looking forward, continued advances in structural biology, live-cell imaging, single-molecule approaches, and genome-wide functional analyses are expected to provide deeper insights into the molecular mechanisms of the SMC5/6 complex. These technologies will facilitate a more comprehensive understanding of how SMC5/6 contributes to DNA replication, DNA repair, chromosome organization, and antiviral restriction in different cellular contexts. In addition, increasing interest in genome instability disorders and virus–host interactions may create new opportunities to translate mechanistic discoveries into biomedical applications. A deeper understanding of SMC5/6 function will not only advance fundamental studies of chromosome biology but may also support the development of future diagnostic and therapeutic approaches for genome instability-associated diseases and viral infections.

## Figures and Tables

**Table 2:** SMC5/6 Complex Subunits Associated with Hereditary Developmental Disorders: Protein Functions, Clinical Phenotypes and Molecular Pathogenic Defects.

Gene	Protein Function	Associated Disorder	Major Clinical Features	Cellular/Molecular Defects
NSMCE2 (NSE2/MMS21) [5]	SUMO E3 ligase subunit	Primordial dwarfism syndrome	Primordial dwarfism; Microcephaly; Developmental delay; Metabolic and endocrine abnormalities	Loss of SUMOylation activity; Replication stress; Chromosomal instability; Defective homologous recombination repair
NSMCE3 (NSE3) [80]	Core subunit involved in DNA binding and complex stability	Lung disease; Immunodeficiency and Chromosome breakage Syndrome (LICS)	Severe adaptive immunodeficiency; Recurrent respiratory infections; Progressive pulmonary disease	Destabilization of the SMC5/6 complex; Chromosome breakage; Increased DNA damage sensitivity
SMC5 [48]	Core ATPase subunit	Atelís syndrome (ATS)	Microcephaly; Short stature; Developmental delay; Congenital heart defect; Hematological abnormalities	Replication stress; Chromosome breakage; Segmented chromosomes; Mosaic variegated hyper-ploid
SLF2(NSE6) [48]	Recruitment factor that targets the SMC5/6 complex to damaged chromatin	Atelís syndrome (ATS)	Microcephaly; Growth retardation; Learning disabilities; Congenital anomalies	Impaired SMC5/6 recruitment; Defective replication Stress response; Chromosome instability

## Data Availability

The mutation data for all subunits of the SMC5/6 complex used in this study were obtained from the cBioPortal for Cancer Genomics database. cBioPortal is an open-access, manually curated public database that integrates multidimensional cancer genomics data and corresponding clinical information from a wide range of human malignancies, enabling the exploration and analysis of cancer-associated genomic alterations. The platform was originally developed by Memorial Sloan Kettering Cancer Center (MSK) and is currently maintained through the collaborative efforts of multiple international research institutions, including MSK, Dana-Farber Cancer Institute, and Princess Margaret Cancer Centre. The database is publicly accessible at https://www.cbioportal.org/ (accessed on 27 August 2026).

## Abbreviations

The following abbreviations are used in this manuscript:

ATP	adenosine triphosphate
BTR	BLM–TopoIIIα–RMI1/2 complex
CAP	chromosome-associated protein
dsDNA	double-stranded DNA
FANCD2	Fanconi anemia group D2 protein
HEAT	Huntingtin, elongation factor 3, protein phosphatase 2A, and TOR1
HR	homologous recombination
KITE	kleisin-interacting tandem winged-helix element
Mms21	methyl methanesulfonate sensitivity protein 21
Mph1	DNA helicase Mph1
NIPBL	Nipped-B-like protein
NPC	nuclear pore complex
Nse	non-SMC element
PDS5	precocious dissociation of sisters protein 5
Rad21	DNA repair protein Rad21
Rad51	RAD51 recombinase
Rad59	RAD59 recombination mediator
RPA	replication protein A
SA1/SA2	stromal antigen 1/2
Scc1	sister chromatid cohesion protein 1
SMC	Structural Maintenance of Chromosomes
ssDNA	single-stranded DNA
STR	Sgs1–Top3–Rmi1 complex
SUMO	small ubiquitin-like modifier
Ub	Ubiquitin
HBV	Hepatitis B virus
HPV	Human papillomavirus
HSV	Herpes simplex virus
HIV-1	Human immunodeficiency virus 1
AAV	Adeno-associated virus
EBV	Epstein–Barr virus
KSHV	Kaposi sarcoma-associated herpesvirus
NSMCE	Non-SMC element; Nse refers to the corresponding yeast subunit nomenclature
NSMCE1-4A	SMC5/6 auxiliary subunits; orthologs of yeast Nse1-Nse4
SIMC1	Adaptor protein; paralog of SLF1, functional ortholog of yeast Nse5
SLF2	Adaptor protein; functional ortholog of yeast Nse6
Mms21:	Methyl methanesulfonate sensitivity protein 21, synonymous designation of yeast Nse2
SLF1	Adaptor protein; paralog of SIMC1, functional ortholog of yeast Nse5
